# Identification of Circulating Exosomal miR-101 and miR-125b Panel Act as a Potential Biomarker for Hepatocellular Carcinoma

**DOI:** 10.1155/2021/1326463

**Published:** 2021-12-27

**Authors:** Li Sun, Mu Xu, Guoying Zhang, Lin Dong, Jie Wu, Chenchen Wei, Kexin Xu, Lu Zhang

**Affiliations:** ^1^Laboratory Medicine Center, the Second Affiliated Hospital, Nanjing Medical University, Nanjing, Jiangsu, China; ^2^Department of Clinical Laboratory, Nanjing First Hospital, Nanjing Medical University, Nanjing, Jiangsu, China; ^3^Department of Clinical Laboratory, Nanjing Integrated Traditional Chinese and Western Medicine Hospital, Nanjing University of Traditional Chinese Medicine, Nanjing, Jiangsu, China; ^4^Cancer Medical Center, the Second Affiliated Hospital, Nanjing Medical University, Nanjing, Jiangsu, China

## Abstract

**Background:**

Hepatocellular carcinoma (HCC) is one of the most common cancers worldwide with high mortality, and there is an urgent need of new diagnosis measures. This study is aimed at investigating whether circulating exosomal miRNAs could act as biomarkers for the diagnosis of HCC.

**Methods:**

A four-stage strategy was adopted in this study. Candidate miRNA was selected by comprehensive analysis of four GEO datasets and TCGA database. The expression of candidate miRNAs in serum exosomal samples were examined through qRT-PCR. The diagnostic utility of the final validated miRNAs was examined by receiver operating characteristic (ROC) curve analysis.

**Results:**

After synthetical analysis of four GEO datasets, six miRNAs were selected as candidates due to their higher differential fold change. miR-101 and miR-125b were selected as candidate miRNAs to further investigate their potential as biomarkers for HCC due to their differential fold change and their influence on overall survival based on the TCGA database. As a result, miR-101 and miR-125b expressions were remarkably downregulated in both tissues and serum exosomes of patients with HCC. The area under the ROC curves (AUCs) of circulating exosomal miR-101 and miR-125b were 0.894 (95% CI, 0.793–0.994) and 0.812 (95% CI, 0.675–0.950), respectively. The combination of the two miRNAs presented higher diagnostic utility for HCC (AUC = 0.953).

**Conclusion:**

The exosomal miR-101 and miR-125b panel in the serum may act as a noninvasive biomarker for HCC detection.

## 1. Introduction

Hepatocellular carcinoma (HCC) is one of the most common cancers worldwide and represents a major cause of cancer-related death [1]. Most patients were diagnosed in late stage, at which point they missed the perfect time for surgical treatment and the efficacy of chemotherapy and radiotherapy is limited. Traditional imaging techniques are widely used for HCC screening; however, they are less efficient for the detection of early HCC. Moreover, analysis of alpha-fetoprotein (AFP) as one of the most important serum tumor biomarkers for HCC is still not satisfactory for the sensitivity and specificity of HCC diagnosis [2, 3]. Because the symptoms of patients with early HCC are not obvious, approximately one-third of patients have normal serum AFP levels, and some patients with benign liver disease even have elevated AFP levels. Therefore, novel biomarkers for the early detection of HCC are urgently needed.

MicroRNAs (miRNAs) are short (approximately 22 nucleotides) single-stranded noncoding RNAs that cause either translational inhibition or degradation of target gene molecules by binding to the 3′UTR [4, 5]. The expression profiles of microRNAs in cancerous tissues are significantly different from those in adjacent normal tissues, thus indicating their potential role as diagnostic biomarkers. In contrast to most previous studies on miRNA biomarkers, which have focused on tissue specimens, some studies have reported the potential value of circulating miRNAs [6–8]. Our previous study showed that circulating miR-182 has potential as a novel noninvasive biomarker to discriminate patients with colorectal cancer from healthy individuals [9]. Tumor-derived circulating miRNAs can exist stably in plasma or serum due to their ability to resist endogenous ribonuclease activity, extreme pH, and temperature [10]. One explanation for this phenomenon is that miRNAs can be packaged into exosomes and then secreted into peripheral blood by cancer cells [11, 12].

Exosomes, a kind of membrane vesicle, with a size from 30 to 150 nm, are released into the extracellular environment when multivesicular bodies fused with cell membrane. Exosomes are remarkably stable in body fluids, and they can carry various contents from donor cells to recipient cells, including proteins, lipids, DNA, and RNA [13, 14]. Emerging evidence has demonstrated that exosomes and their delivered miRNAs have potential use as novel potential biomarkers in various cancers, such as HCC [15], pancreatic ductal adenocarcinoma [16], glioblastoma [17], prostate cancer [18], colorectal cancer [19], and breast cancer [20]. However, many circulating miRNAs that have been identified as potential diagnostic biomarkers of HCC were not selected through high-throughput screening; thus, other miRNAs that may have a higher diagnostic value were ignored. The aim of this study was to clarify the potential of circulating exosomal miRNA as a biomarker for the diagnosis of patients with HCC based on the Gene Expression Omnibus (GEO) database, the Cancer Genome Atlas (TCGA) database, and our experiments.

## 2. Materials and Methods

### 2.1. Study Design

**Figure 1:**
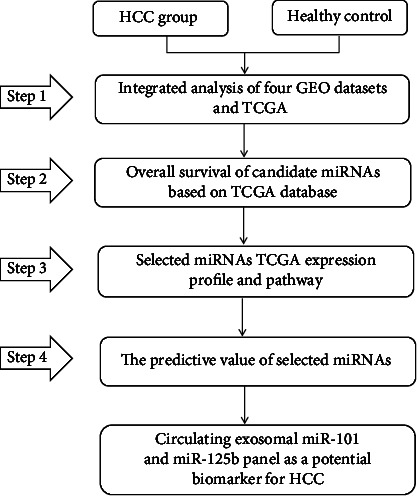
Study design of the workflow. Flowchart showing the detailed process of how the circulating exosomal miR-101 and miR-125b panel was selected and validated in HCC.

This study included four phases.  Figure 1 shows the overall workflow of this study. In the discovery phase, we conducted a synthetical analysis of miRNA expression using four GEO datasets. The selected miRNAs were validated and further investigated in the TCGA datasets due to the differential fold-change expression and their influence on overall survival. Subsequently, the expression of the selected serum exosomal miRNAs (miR-101 and miR-125b) was detected by qRT-PCR in 40 samples (20 healthy subjects and 20 patients with preoperative HCC).

### 2.2. Analysis of GEO and TCGA Databases

GEO datasets on miRNA expression in HCC were searched using the keywords “microRNA” and “hepatocellular carcinoma.” The differentially expressed miRNAs were analyzed using the online tool GEO2R. The GEO datasets were selected for the analysis provided that they met the following inclusion criteria: (1) The object of the study was miRNA expression between HCC and nontumor liver tissues. (2) The method of the study was noncoding RNA profiling by array or genome-wide miRNAs screening. (3) Among these deregulated miRNAs, those with *P* < 0.05 were included. The miRNA expression profile of various human cancers and adjacent normal tissues was collected from the TCGA data online analysis tool. The pathway of miRNA of different cancers and some signaling pathways were obtained using the online TCGA tool.

### 2.3. Study Population and Sample Preparation

HCC patients and healthy controls were recruited from the Second Affiliated Hospital of Nanjing Medical University. None of the recruited patients had received chemotherapy or radiation therapy before specimens were collected. Healthy controls were collected from volunteers participating in the physical examination. Original serum samples were prepared by centrifugation at 4,000 rpm for 10 min from blood samples which were collected in serum separator tubes. The serum was then divided into multiple aliquots of 500 *μ*L and stored at −80°C prior to this study. All participants provided written informed consent. This study was approved by the Ethics Committee of the Second Affiliated Hospital of Nanjing Medical University.

### 2.4. Exosome Isolation

Exosomes were isolated using the Total Exosome Isolation Kit (Invitrogen, USA) according to the manufacturer's instruction as previously described [21–23]. The serum was thawed in a 25°C water bath and then subjected to centrifugation at 2,000 × g for 30 min to remove possible residual cells debris. Next, 500 *μ*L serum was mixed with 100 *μ*L Total Exosome Isolation Reagent, followed by incubation at 4°C for 30 min and centrifugation at 10,000 × g for 10 min at room temperature. The exosome pellet dissolved in 100 *μ*L phosphate-buffered saline (PBS) was ready for RNA isolation.

### 2.5. RNA Isolation from Serum Exosomes

Total exosome RNA was extracted using TRIzol reagent (Invitrogen, USA) following the manufacturer's instructions. Cel-miR-39 (RiboBio, Guangzhou, China) was added to each sample at a final concentration of 10^−4^ pmol/*μ*L acting as the external reference. The extracted RNA was eluted with 14 *μ*L of nucleic acid-free water. The RNA quality and quantity were determined using the spectrophotometer OD-1000+ (OneDrop, USA).

### 2.6. qRT-PCR for miRNA Quantitation

Reverse transcription and qRT-PCR for miR-101, miR-125b, and external reference miR-39 were performed using Hairpin-it™ miRNA RT-PCR Quantitation Kit (GenePharma, China) according the manufacturer's instructions. The reactions were initiated with denaturation at 95°C for 3 min, followed by 40 cycles of 95°C for 15 s and 62°C for 34 s. The relative expression of miR-101 and miR-125b was calculated using the 2^–△△Ct^ method (ΔCT = CT miRNA − CT reference).

### 2.7. Statistical Analysis

The different expressions of miRNAs among groups were determined using the Mann–Whitney unpaired test or paired *t*-test. We next applied the receiver operating characteristic (ROC) curve to evaluate the predictive value of the selected miRNAs for HCC. All of the statistical analyses were performed using SPSS 25.0 (IBM) and GraphPad 8.0 (GraphPad Software). Each assay was performed at least three times. The data were expressed as mean ± SD. A *P*  value < 0.05 was considered to be statistically significant.

## 3. Results

### 3.1. Integrated Analysis of Four GEO Datasets and TCGA Database Identified Five miRNAs with Significantly Dysregulated Expression in HCC Tissues

**Figure 2:**
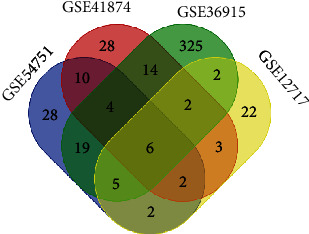
Six consistently dysregulated microRNAs were selected to further explore their expression and potential diagnostic utility after an integrated analysis of four GEO datasets. Notes: Venn graph of the integrated analysis of GSE54751, GSE41874, GSE36915, and GSE12717. Six miRNAs: miR-101, miR-125b, miR-18a, miR-224, miR-378, and miR-424.

**Figure 3:**
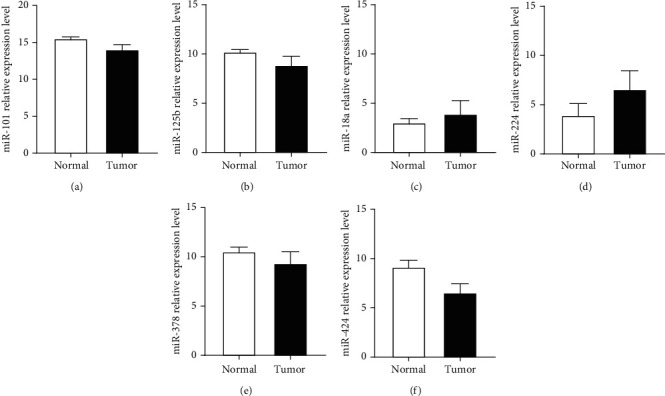
Five miRNAs were selected to further explore their potential diagnostic utility by analysis of TCGA databases. Notes: (a–f) only five miRNAs (miR-101, miR-125b, miR-224, miR-378, and miR-424) were significantly deregulated in HCC tissues using the TCGA database. Fold change < 1 in miR-18a.

First, we manually found four GEO datasets (GSE54751, GSE41874, GSE36915, and GSE12717), which were used to conduct a comprehensive comparative analysis of miRNA expression in HCC and normal hepatic tissues. After merging these datasets, six consistently dysregulated microRNAs were identified, namely, miR-101, miR-125b, miR-18a, miR-224, miR-378, and miR-424 (Figure 2). Next, we further validated the six dysregulated microRNAs in the TCGA HCC dataset (375 HCC tissues, 50 normal hepatic tissues). Only five miRNAs, namely, miR-101, miR-125b, miR-224, miR-378, and miR-424, were significantly deregulated in HCC tissues (Figure 3).

### 3.2. Expressions of Tissular miR-101 and miR-125b Were Significantly Associated with Prognosis of Patients with HCC

**Figure 4:**
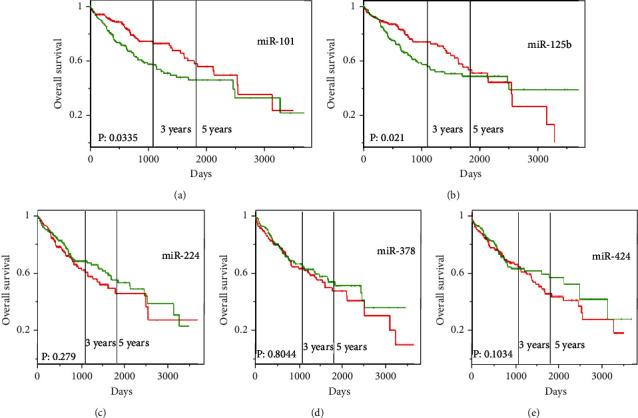
Prognostic significance of miR-101 and miR-125b in HCC from ProgmiR. The cohort was stratified by the median expression level of the five selected miRNAs. (a) The influence of miR-101 expression on overall survival in HCC (HR = 0.8, 0.64–0.98, *P* = 0.0335). (b) The influence of miR-125b expression on overall survival in HCC (HR = 0.92, 0.85–0.99, *P* = 0.021). (c) The influence of miR-224 expression on overall survival in HCC (HR = 1.05, 0.96–1.16, *P* = 0.279). (d) The influence of miR-378 expression on overall survival in HCC (HR = 1.02, 0.89–1.16, *P* = 0.8044). (e) The influence of miR-424 expression on overall survival in HCC (HR = 1.15, 0.97–1.36, *P* = 0.1034). The red line represents high expression, and the green line represents low expression.

**Figure 5:**
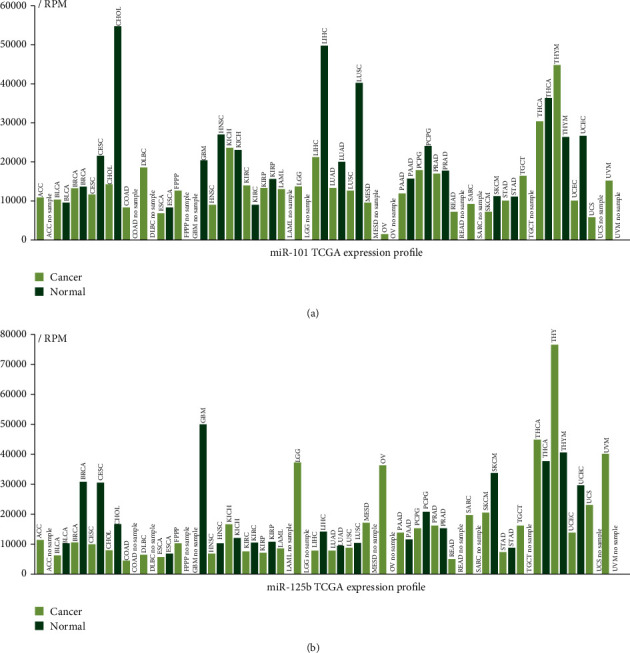
Expression profile of miR-101 and miR-125b from TCGA. (a) miR-101 was downregulated in liver hepatocellular carcinoma tissues compared with normal tissues. (b) miR-125b was downregulated in liver hepatocellular carcinoma tissues compared with normal tissues. Abbreviations: LIHC: liver hepatocellular carcinoma.

Five selected miRNAs, namely, miR-101, miR-125b, miR-224, miR-378, and miR-424, were associated with overall survival by online TCGA analysis. Only two miRNAs, namely, miR-101 and miR-125b, were significantly associated with the prognosis of patients (Figure 4). Subsequently, we investigated the TCGA expression profile of the two miRNAs in different tumor tissues and nontumor tissues (Figure 5). Considering the varying expressions of miR-101 and miR-125b between cancerous tissue and normal hepatic tissues, as well as their expression being associated with the prognosis of patients with HCC, we further investigated whether the dysregulation of miR-101 and miR-125b in peripheral blood exosomes serves as a diagnostic marker for HCC. We also found different pathways of miR-101 and miR-125b, as shown in Tables 1 and 2.

### 3.3. Circulating Exosomal miR-101 and miR-125b Panel Acts as a Novel Potential Diagnostic Biomarker for HCC

**Figure 6:**
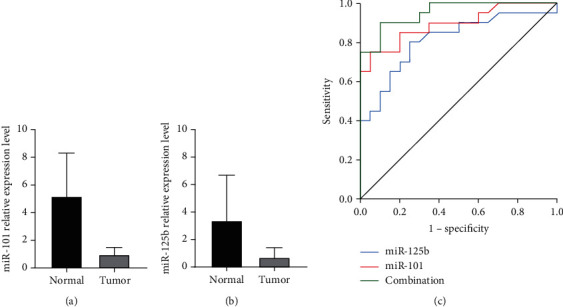
The diagnostic utility of circulating exosomal miR-101 and miR-125b was further validated to be potential biomarkers for HCC. (a) Circulating exosomal miR-101 was downregulated in patients with HCC compared to healthy controls. Data are presented as mean ± SD. *P* < 0.05. (b) Circulating exosomal miR-125 was downregulated in patients with HCC compared to healthy controls. Data are presented as mean ± SD. *P* < 0.05. (c) The AUC was 0.894 (95% CI, 0.793–0.994) for miR-101, 0.812 (95% CI, 0.675–0.950) for miR-125b, and 0.953 (95% CI, 0.895–1.000) for the miRNA combination.

To further validate the potential diagnostic role of circulating exosomal miR-101 and miR-125b, an independent cohort composed of 20 healthy subjects and 20 patients with preoperative HCC was examined. There was no statistically significant difference in age and sex between healthy subjects and patients with HCC. The relative expression levels of miR-101 and miR-125b were significantly downregulated in the HCC serum exosome samples compared to the healthy controls (Figures 6(a) and 6(b)). ROC curve analysis was performed to assess the potential diagnostic role of miRNAs. Individually, the AUC was 0.894 (95% CI, 0.793–0.994) for miR-101, 0.812 (95% CI, 0.675–0.950) for miR-125b, and 0.953 (95% CI, 0.895–1.000) for the miRNA combination (Figure 6(c)).

## 4. Discussion

The aim of this study was to discover specific exosomal miRNAs in peripheral blood as noninvasive biomarkers for HCC detection. Emerging evidence has showed that cancer cells secret exosomes containing miRNAs into various body fluids, including peripheral blood, to transport signals from donor cells to recipient cells [24, 25]. Our study demonstrated that exosomal miR-101 and miR-125b in the serum are potential diagnostic biomarkers of HCC.

We performed a comprehensive comparative analysis of miRNA expression profiles based on four GEO datasets and TCGA database and discovered five consistently dysregulated microRNAs (miR-101, miR-125b, miR-224, miR-378, and miR-424) in patients with HCC. Considering that the poor outcomes of patients with HCC mainly result from the advanced stage of diagnosis, we then analyzed the TCGA database and found that two miRNAs (miR-101 and miR-125b) were significantly associated with the prognosis of patients with HCC. Mortality could be reduced if patients with HCC could be diagnosed at an early stage and treated before malignancy develops into an advanced stage [26]. Thus, new diagnosis strategies for HCC are urgently needed.

Recently, noncoding RNAs have attracted much attention as potential diagnostic and prognostic biomarkers in human cancers due to their involvement in vital oncogenic processes such as proliferation, migration, differentiation, angiogenesis, and apoptosis. Emerging lines of evidence have shown that the mutational spectrum and dysregulated expression of noncoding RNA genes, such as circRNAs, lncRNAs, and miRNAs are closely associated with the development and progression of various cancers [27–33]. Barbagallo and colleagues reported that circSMARCA5 regulated the interaction with SRSF1 and some related downstream effects on glioblastoma multiforme cell migration and angiogenic potential through in vitro and in silico characterization of its GAUGAA motif sequences [30]. Moreover, serum extracellular vesicle-derived circSMARCA5 has also been reported as a good diagnostic biomarker for GBM [31]. lncRNA LINC00518, under potential transcriptional control by MITF, regulates an RNA–RNA network promoting cancer-related processes [32]. miRNA-802 inhibits the growth and metastatic-related phenotypes of cervical cancer cell through targeting MYLIP [33]. Besides, Cheng and colleagues reported the establishment of a ceRNA network jointly participated by circRNAs and lncRNAs for the first time. The ceRNA network of 6 circRNAs, 32 lncRNAs, 8 miRNAs, and 6 mRNAs was established as novel prognostic markers for acute myeloid leukemia, which has important guiding significance for the clinical diagnosis, treatment, and further scientific research [34].

Exosomes also aroused our intrigue due to their stability in peripheral blood. Mounting reports have indicated that exosomal miRNAs are promising biomarkers for cancers. Yokota and colleagues reported that serum exosomal miR-638 is a prognostic marker of HCC via downregulation of endothelial cell VE-cadherin and ZO-1 [15]. Moreover, Wang and colleagues suggested that serum exosomal miRNA-1226-3p is a potential biomarker for diagnosing and predicting the tumor invasion or metastases of pancreatic ductal adenocarcinoma [16]. Furthermore, Shen and colleagues demonstrated that cancer-derived exosomal miR-7641 may serve as a promising noninvasive diagnostic biomarker and potential targetable candidate in breast cancer treatment [20]. We confirmed that circulating exosomal miR-101 and miR-125b panel served as a novel promising biomarker for diagnosing HCC because of its reliable ability to differentiate patients with HCC from normal controls.

Here, we found that serum exosomal miR-101 and miR-125b expression levels were significantly downregulated in patients with HCC and demonstrated that the exosomal miR-101 and miR-125b panel acted as a promising biomarker for the diagnosis of HCC. However, the sample size was relatively small, and more prospective studies with larger sample numbers are needed to clarify the clinical value of this biomarker.

Several studies have investigated the role of miR-101 and miR-125b in cancers. Wu et al. demonstrated that miR-101 promotes nasopharyngeal carcinoma cell apoptosis through inhibiting the Ras/Raf/MEK/ERK signaling pathway [35]. The data generated by Zhang et al. support the function of miR-101 as a tumor suppressor in osteosarcoma via the downregulation of BCL6 [36]. In addition, miR-125b loss was reported to activate the HIF1*α*/pAKT loop, contributing to HCC resistance to transarterial chemoembolization [37]. Based on TCGA, we found that the miR-101 pathway was associated with cancers, including melanoma, renal cell carcinoma, colorectal cancer, and the p53 signaling pathway. The miR-125b pathway has been found to be associated with cancers, such as bladder cancer, melanoma, prostate cancer, pancreatic cancer, and colorectal cancer, and several pathways, including the ErbB signaling pathway, the VEGF signaling pathway, and the p53 signaling pathway. It has also been shown that miR-125b is associated with hepatitis B and has been targeted with genes that include IL6, DDX3X, STAT2, STAT3, SMAD4, CDKN1B, MAP3K1 and so on from our results. However, the effect of miR-101 and miR-125b has not been investigated in HCC. Therefore, the pathologic effect of miR-101 and miR-125b should be further explored by conducting gain and loss of function assays both in vitro and in vivo.

In conclusion, we demonstrated that the serum exosomal miR-101 and miR-125b panel is a novel potential diagnostic biomarker of HCC.

## Figures and Tables

**Table 1:** miR-101 pathway.

KEGG	kegg_dscp	Gene	*p* value	Possibility
ko05218	Melanoma	IGF1R, CCND1, CDKN1A, NRAS, MET, MAP2K1, CDK6, MITF	2.83*E* − 54	0.017683847
ko05211	Renal cell carcinoma	MET, JUN, RAC1, NRAS, RAP1B, VHL, MAP2K1, CRK, EP300, PAK3	3.58*E* − 69	0.015166268
ko05210	Colorectal cancer	TGFBR2, TGFBR1, CASP3, RAC1, CCND1, BIRC5, FOS, JUN, GSK3B, MAP2K1, RHOA, MSH2	3.52*E* − 62	0.025836046
ko04115	p53 signaling pathway	CCND2, CASP3, CDK1, CCND1, CDKN1A, ATM, CCNG1, ZMAT3, PPM1D, RRM2, CDK6	8.02*E* − 77	0.014138969
ko05220	Chronic myeloid leukemia	TGFBR2, TGFBR1, CCND1, CDKN1A, NRAS, MAP2K1, RUNX1, CDK6, CRK	3.99*E* − 74	0.010847935
ko04520	Adherens junction	TGFBR2, IGF1R, TJP1, SSX2IP, RAC1, VCL, PTPRJ, FYN, TGFBR1, MAP3K7, PVRL1, MET, PVRL2, NLK, PTPN1, EP300, RHOA	2.42*E* − 66	0.039034869

The detailed miR-101 pathway was associated with different cancers and signaling pathways and their targeted genes based on the TCGA database.

**Table 2:** miR-125b pathway.

KEGG	kegg_dscp	Gene	*p* value	Possibility
ko00514	Other types of O-glycan biosynthesis	B4GALT1, B3GALTL, B4GALT3, FUT7, OGT	3.12*E* − 47	0.011742378
ko05219	Bladder cancer	ERBB2, CDKN2A, TP53, E2F2, NRAS, RAF1, KRAS	4.73*E* − 51	0.017759706
ko05218	Melanoma	CDKN2A, TP53, E2F2, NRAS, CDK6, FGFR1, RAF1, KRAS	4.78*E* − 57	0.017526623
ko05217	Basal cell carcinoma	CTNNB1, TCF7, FZD4, AXIN2, AXIN1, SMO, PTCH2, TP53	2.73*E* − 60	0.015535401
ko05216	Thyroid cancer	TCF7, TP53, NRAS, RET, CTNNB1, TPR, KRAS	2.01*E* − 42	0.026347694
ko05215	Prostate cancer	ERBB2, TCF7, TP53, E2F2, CDKN1B, NRAS, CREB1, CTNNB1, FGFR2, IKBKG, BCL2, KRAS, RAF1, FGFR1	5.06*E* − 89	0.017111648
ko05214	Glioma	CDKN2A, TP53, E2F2, NRAS, CDK6, RAF1, KRAS	1.32*E* − 62	0.011336943
ko05213	Endometrial cancer	ERBB2, TCF7, TP53, AXIN2, AXIN1, NRAS, CTNNB1, ELK1, RAF1, KRAS	3.43*E* − 54	0.028333084
ko05212	Pancreatic cancer	ERBB2, CDKN2A, STAT3, RAC3, SMAD4, E2F2, ARHGEF6, IKBKG, CDK6, TP53, RAF1, KRAS	1.26*E* − 71	0.021190757
ko05210	Colorectal cancer	TCF7, TP53, RAC3, AXIN1, SMAD4, JUN, CTNNB1, BCL2, KRAS, RAF1, AXIN2	9.87*E* − 68	0.020677737
ko00603	Glycosphingolipid biosynthesis-globo series	A4GALT, GBGT1, NAGA	6.78*E* − 26	0.017862543
ko00601	Glycosphingolipid biosynthesis-lacto and neolacto series	B3GNT5, GCNT2, FUT7, FUT3, B4GALT1, B4GALT3	1.93*E* − 39	0.023770631
ko05332	Graft-versus-host disease	IFNG, IL6, CD28, PRF1	2.84*E* − 31	0.019578864
ko04722	Neurotrophin signaling pathway	TP53, NRAS, MAP3K1, JUN, KIDINS220, TP73, NTRK3, PRDM4, ABL1, MAP2K7, BCL2, IRAK3, SORT1, CRK, IRAK1, RAF1, MAPKAPK2, KRAS	4.54*E* − 105	0.018112601
ko04012	ErbB signaling pathway	ERBB2, ERBB3, EIF4EBP1, JUN, NRAS, PAK3, ELK1, MAP2K7, ABL1, CRK, ABL2, RAF1, KRAS	3.19*E* − 81	0.018457916
ko04370	VEGF signaling pathway	RAC3, NRAS, PXN, RAF1, MAPKAPK2, KRAS	4.13*E* − 55	0.011490893
ko04115	p53 signaling pathway	CDKN2A, PMAIP1, PPM1D, TP73, IGFBP3, PERP, CDK6, TP53, BBC3, SERPINE1	2.54*E* − 83	0.011151405
ko03013	RNA transport	NUP133, EIF2B5, EIF5, EIF5B, EIF2S3, XPO1, RBM8A, NDC1, EIF4EBP1, NUP210, RANBP2, KPNB1, RNPS1, ACIN1, NUP93, CASC3, TPR, EIF4E, UBE2I, RPP14, NUP37, POP7, NUP155, NUP205	5.94*E* − 151	0.013146709
ko05223	Non-small-cell lung cancer	ERBB2, CDKN2A, TP53, E2F2, NRAS, RASSF5, CDK6, RAF1, KRAS	1.24*E* − 59	0.019436868
ko05220	Chronic myeloid leukemia	CDKN2A, TP53, E2F2, SMAD4, CDKN1B, NRAS, IKBKG, CDK6, ABL1, CRK, RAF1, KRAS	1.26*E* − 71	0.021190757
ko05221	Acute myeloid leukemia	TCF7, STAT3, NRAS, CEBPA, EIF4EBP1, IKBKG, RAF1, KRAS	3.19*E* − 68	0.011822332
ko04520	Adherens junction	ERBB2, TCF7, RAC3, WASF2, SMAD4, VCL, CTNNB1, INSR, PTPN1, FGFR1	8.96*E* − 85	0.010727212
ko05161	Hepatitis B	IL6, DDX3X, STAT2, STAT3, SMAD4, CDKN1B, MAP3K1, JUN, CREB1, E2F2, ELK1, IKBKG, CDK6, BCL2, TP53, MAVS, RAF1, NRAS, KRAS	2.93*E* − 129	0.01254609
ko05162	Measles	MAVS, CD28, STAT3, STAT2, IFNG, CDKN1B, ADAR, IL13, TNFAIP3, IL6, DOK1, TP73, CDK6, TP53, BBC3, IRAK1, TACR1	3.92*E* − 127	0.010775044

The detailed miR-125b pathway was associated with different cancers and signaling pathways and their targeted genes based on the TCGA database.

## Data Availability

Data is available at Digital Object Identifier 10.4121/16621162 (https://data.4tu.nl/account/home).
